# Quantum based effects of therapeutic nuclear magnetic resonance persistently reduce glycolysis

**DOI:** 10.1016/j.isci.2022.105536

**Published:** 2022-11-09

**Authors:** Viktoria Thöni, David Mauracher, Anil Ramalingam, Birgit Fiechtner, Adolf Michael Sandbichler, Margit Egg

**Affiliations:** 1 Institute of Zoology, University Innsbruck, Innsbruck, Tyrol A-6020, Austria

**Keywords:** Therapy, Nuclear magnetic resonance, Cell biology

## Abstract

Electromagnetic fields are known to induce the clock protein cryptochrome to modulate intracellular reactive oxygen species (ROS) via the quantum based radical pair mechanism (RPM) in mammalian cells. Recently, therapeutic Nuclear Magnetic Resonance (tNMR) was shown to alter protein levels of the circadian clock associated Hypoxia Inducible Factor-1α (HIF-1α) in a nonlinear dose response relationship. Using synchronized NIH3T3 cells, we show that tNMR under normoxia and hypoxia persistently modifies cellular metabolism. After normoxic tNMR treatment, glycolysis is reduced, as are lactate production, extracellular acidification rate, the ratio of ADP/ATP and cytosolic ROS, whereas mitochondrial and extracellular ROS, as well as cellular proliferation are increased. Remarkably, these effects are even more pronounced after hypoxic tNMR treatment, driving cellular metabolism to a reduced glycolysis while mitochondrial respiration is kept constant even during reoxygenation. Hence, we propose tNMR as a potential therapeutic tool in ischemia driven diseases like inflammation, infarct, stroke and cancer.

## Highlights

• Therapeutic nuclear magnetic resonance affects HIF-1α expression
• tNMR persistently reduces hypoxia induced glycolysis in mammalian cells
• tNMR prevents increased mitochondrial respiration after acute hypoxia
• tNMR alters cellular ROS signature comparable to that of the radical pair mechanism

## Graphical abstract

**Figure:**
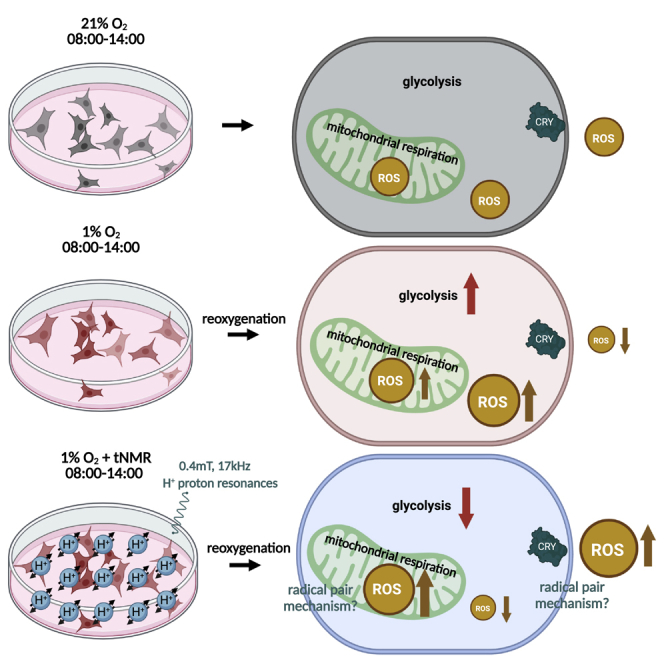

## Introduction

The discovery of the core clock protein cryptochrome (CRY) as a receptor molecule for magnetic sensing in migrating animals gave rise to the question of a potential impact of man-made electromagnetic fields (EMFs) on the circadian clocks of somatic cells.^1^^,^^2^^,^^3^^,^^4^ Although recent studies actually demonstrate that cellular clocks are affected by weak electromagnetic fields, knowledge on the underlying mechanisms or even on the physiological consequences is still scarce. Because the circadian system is intimately intertwined with the hypoxic signaling pathway,^5^^,^^6^ the idea to study the associated hypoxic signaling pathway in response to EMFs, in our case a Radiofrequency (RF) EMF, was obvious. Only recently, we were able to show that the expression of Hypoxia-inducible factor-1 alpha (Hif-1α) indeed follows a nonlinear dose response relationship at the level of mRNA and protein in response to different treatment durations of therapeutic Nuclear Magnetic Resonance (tNMR, MBST® open system 350, MedTec Medizintechnik GmbH, Wetzlar, Germany, 0.4mT and 17 kHz).^4^ We therefore decided to address the physiological consequences of the tNMR modulated Hif-1α levels observed and set out to investigate basic cellular metabolism such as glycolysis, mitochondrial respiration, cellular redox state and reactive oxygen species (ROS) signaling in response to tNMR. We performed the measurements after tNMR treatment under atmospheric oxygen saturation, which is actually hyperoxic for cells in culture,^7^ and after tNMR treatment under acute hypoxia of 1% O_2_ for six hours. Because hypoxic signaling is controlled by Hif-1α and rather well understood in mammalian cells, any potential impact of tNMR should be easier to observe and understand.

HIF-1α is a basic helix-loop-helix-PAS domain transcription factor responsible for oxygen dependent physiological cellular adaptations and oxygen distribution in cells, tissues and organisms. To form an active transcription factor HIF-1α heterodimerizes with its β subunit HIF-1β, the latter of which is constitutively expressed. The alpha subunit, in turn, though being constitutively expressed as well, undergoes constant degradation under normoxic conditions. This process of degradation under sufficient O_2_ availability is initiated by the O_2_-dependent hydroxylation of proline residues 402 and/or 564 of the protein through prolyl hydroxylase domain protein 2 (PHD2). PDH2 consequently promotes the binding of the von Hippel-Lindau (VHL) protein, leading to subsequent ubiquitination of the protein and a final degradation by the 26S proteasome. In parallel, the asparagine residue 803 is hydroxylated by factor inhibiting HIF-1 (FIH-1), a process which is as well dependent on sufficient O_2_ and which blocks the binding of the 300 kD coactivator protein (p300) and CREB binding protein (CBP), leading to a further inhibition of HIF-1α activity. Under conditions of low oxygen concentrations, the activities of hydroxylases are inhibited. Because of this essential role in oxygen dependent metabolism, HIF-1α plays central roles in a variety of pathophysiological conditions, such as cardiovascular disease,^8^^,^^9^ all forms of ischemic conditions,^10^^,^^11^ osteoarthritis^12^^,^^13^ and tumor biology.^14^^,^^15^ Hence, much effort has been laid on targeting HIF-1α as therapeutic option. Pitfalls therein were and still are complex situation-specific outcomes, such as dependence on cell types, in particular tumor cell types and tumor microenvironment,^16^ significance of the temporal application (examples are the ischemia reperfusion injury^17^ or the distinct, often opposing roles of HIF-1α during tumor initiation and during metastatic spread) , as well as the severe side effects in case of rather non-specific pharmacological inhibition.^18^^,^^19^ Radiotherapy for the treatment of cancer has been shown to affect HIF-1 signaling as well, and the latter itself has been shown to be responsible for the often developing radioresistance of tumor cells.^20^^,^^21^

Here, we show that tNMR reduces glycolysis and extracellular acidification while mitochondrial respiration is kept constant and that this effect is even more pronounced when cells experience the tNMR treatment under hypoxia of 1% O_2_ and during subsequent reoxygenation. tNMR, thus, might have a clear potential as therapeutic tool for various pathophysiological ischemic conditions, in which a rewiring of basic cellular metabolism is wanted, such as inflammation, infarct, stroke and cancer.

## Results

### tNMR affects the expression of Hif-1α at the mRNA and protein level under hypoxic conditions

**Figure 1:**
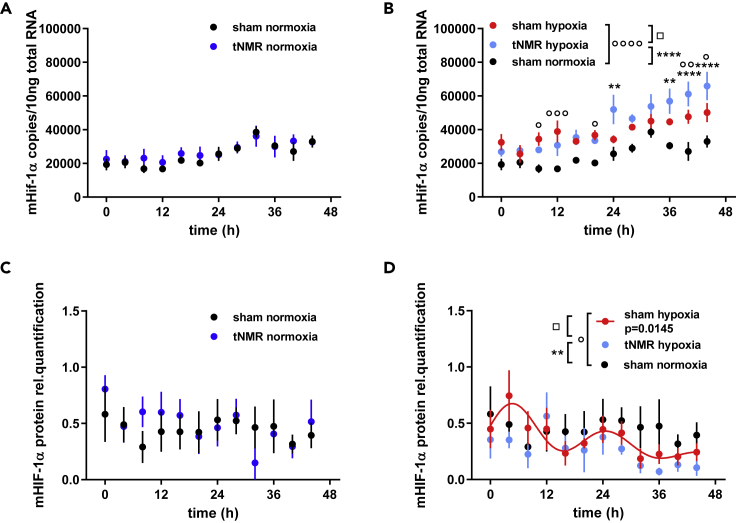
mRNA and protein expression profiles of HIf-1α under normoxia and after 6 h of hypoxic treatment (1% O_2_ and 5% CO_2_), with or without application of tNMR in NIH3T3 cells (A) comparison of mHif-1α mRNA levels between sham (black) and tNMR (blue) treated cells under normoxic conditions. (B) comparison of mHif-1α mRNA levels between sham (black), hypoxic sham (red) and hypoxic tNMR (light blue) treated cells. (C and D) comparison of mHIF-1α protein levels between sham (black) and tNMR (blue) treated cells under normoxic conditions and (D) comparison of mHIF-1α protein levels between sham (black), hypoxic sham (red) and hypoxic tNMR (light blue) treated cells; shown are means ± SEM, n = 4; asterisks mark significant differences between sham and hypoxic tNMR treated cells, squares mark differences between hypoxic sham cells and hypoxic tNMR treated cells and circles mark differences between normoxic and hypoxic sham treated cells, as calculated using two-way ANOVAs (GraphPad Prism 6.0).

In our former publication^4^ we reported the dose dependent nonlinear effects of tNMR on murine Hif-1α mRNA and protein expression in unsynchronized NIH3T3 cells. To explore eventual tNMR induced alterations of cellular metabolism, we now concentrated on the six-hour treatment, screening levels every 4 h, for two whole circadian cycles, using dexamethasone (Dex) synchronized NIH3T3 cells (Experimental setup shown in Figure S1). Under normoxic conditions we did not find significant differences in Hif-1α mRNA and protein expression (Figures 1A and 1C) between sham and tNMR treated cells, which is in contrast to the data observed in unsynchronized cells.^4^ The application of hypoxia (1% O_2_, 5% CO_2_ and 94% N_2_) for 6 h led to significant differences between normoxic control (sham normoxia) and hypoxic treated cells (sham hypoxia and tNMR hypoxia), Figures 1B and 1D). mRNA levels of Hif-1α were increased in hypoxic treated cells, and the increase was higher over the whole second day of sampling. During this period of time, between 24 and 48 h after the treatment, tNMR treated cells exhibited an even more pronounced and significant rise in mRNA levels compared to the solely hypoxia treated cells (Figure 1B). At the level of protein, hypoxic treatment of cells led to a circadian oscillation of HIF-1α, as indicated by the red cosine wave fit to the data (Figure 1D, p = 0.0145). Overall protein amounts of hypoxic treated sham cells appeared to be slightly decreased when compared to normoxic samples. This is not what one would normally expect, knowing that HIF-1α is stabilized under hypoxic conditions. We assume that the treatment with the cortisone derivative Dex might interfere with HIF-1α protein amounts, as reported previously.^22^^,^^23^ The combined treatment of tNMR and hypoxia (tNMR hypoxia) led to significantly altered HIF-1α protein levels, namely a further overall reduction in protein amounts, but also a highly modified temporal expression of the protein (Figure 1D). Differences were most pronounced between hypoxic sham and tNMR treated cells.

### tNMR reduces lactate production and decreases cellular ADP levels under normoxic conditions

**Figure 2:**
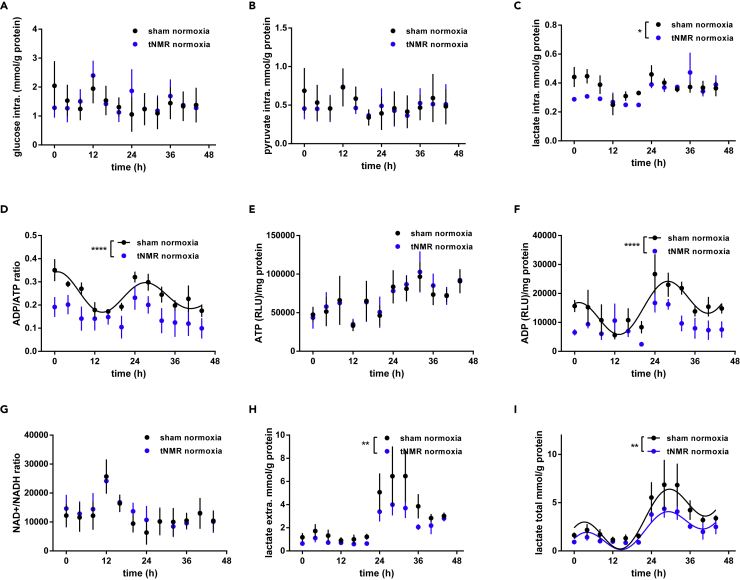
Metabolic signature of synchronized NIH3T3 cells under normoxia, comparison between sham and tNMR treatment (A–I) Levels of (A) glucose, (B) pyruvate, (C) lactate, (D) ADP/ATP ratio, (E) ATP, (F) ADP, (G) NAD+/NADH ratio, as well as (H) extracellular lactate and (I) overall lactate production: comparison between sham (black circles) and tNMR treated (dark blue circles) cells. Shown are means ± SEM, n = 4; asterisks mark significant differences between normoxic sham and tNMR treated cells, as calculated by two-way ANOVAs (GraphPad Prism 6.0).

Because of the differences in Hif-1α expression we set out to investigate metabolites of cellular glycolysis in response to tNMR treatment under normoxic conditions, using the same experimental setup (Figure S1). No differences were found in intracellular glucose and pyruvate levels (Figures 2A and 2B). Intracellular lactate was reduced during the first 24 h of sampling, which is one day after the tNMR treatment (Figure 2C). Most prominent alterations were found in the intracellular ADP/ATP ratio, which was highly reduced in tNMR compared to sham treated cells (Figure 2C). This reduction obviously was due to severely reduced intracellular ADP levels, while ATP stores were equal to control cells (Figures 2E and 2F). No changes were found in the intracellular ratio of NAD^+^/NADH (Figure 2G). In line with the reduced intracellular lactate levels (Figure 2C), extracellular lactate concentrations (Figure 2H) were decreased after tNMR treatment, resulting in reduced overall lactate production (Figure 2I). Hence, among the screened metabolites, only lactate and ADP were significantly affected by tNMR.

### tNMR alters the metabolic signature after hypoxic treatment except for the NAD+/NADH ratio

**Figure 3:**
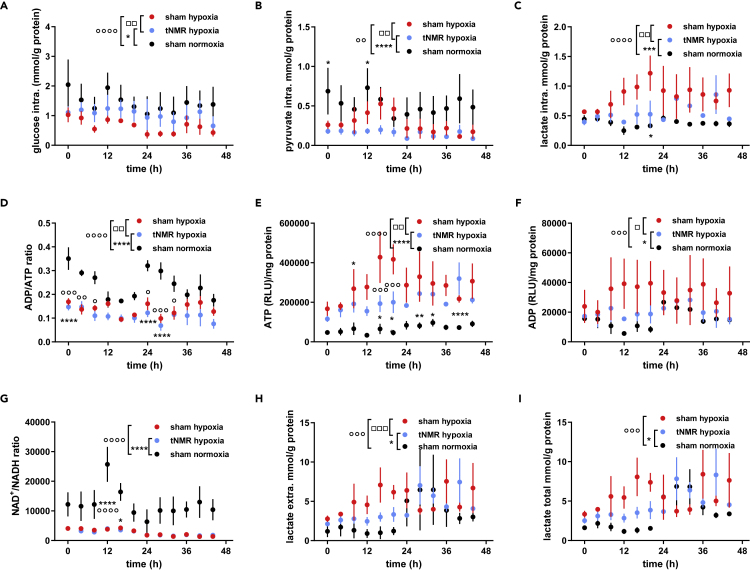
Metabolic signature of synchronized NIH3T3 cells under hypoxic conditions, comparison between sham and tNMR treatment (A–I) Levels of (A) glucose, (B) pyruvate, (C) lactate, (D) ADP/ATP ratio, (E) ATP, (F) ADP, (G) NAD+/NADH ratio, as well as (H) extracellular lactate and (I) overall lactate production after normoxic (black circles), hypoxic (red circles), as well as after combined treatment of tNMR and hypoxia (light blue circles) over two circadian cycles. Shown are means ± SEM, n = 4; open circles mark significant differences between normoxic and hypoxic sham treated cells, asterisks mark significant differences between sham treated normoxic cells and cell treated with tNMR under hypoxic conditions and open squares mark differences between hypoxic sham treated cells and hypoxic tNMR treated cells, calculated by two-way ANOVAs (GraphPad Prism 6.0).

After hypoxic tNMR treatment intracellular glucose levels were reduced compared to normoxic cells (Figure 3A). This commonly known hypoxia induced effect was significantly weakened after tNMR treatment under hypoxic conditions. Intracellular pyruvate, which was as well decreased in hypoxic control cells, appeared to be further decreased after tNMR under hypoxia (Figure 3B). Astonishingly, the further decreased intracellular pyruvate levels did not result in an increased lactate production of tNMR treated cells under hypoxia. On the contrary, the combined treatment drove intracellular lactate levels towards those of normoxic cells, while solely hypoxia treated cells showed increased intracellular lactate levels as expected (Figure 3C). In addition, tNMR under hypoxia further decreased the hypoxia induced decrease of the intracellular ADP/ATP ratio (Figure 3D), by decreasing intracellular ATP levels less than those of ADP (Figures 3E and 3F). After hypoxic conditions intracellular ATP stores were high, while tNMR lead to ATP stores residing in between normoxic and hypoxic cells. Intracellular ADP levels, in turn, were closer to those of normoxic cells. Conclusively, tNMR under hypoxic conditions led to relatively higher ATP versus ADP levels. No differences were found in the intracellular ratio of NAD^+^/NADH between hypoxic treatment and the combined treatment of tNMR and hypoxia, both being equally reduced in comparison to normoxic cells (Figure 3G). As expected, extracellular lactate was highest in hypoxia treated cells, but reduced after the combined treatment tNMR and hypoxia (Figure 3H), though still higher than that of normoxic control cells. Total production of lactate was highest in hypoxic cells, lower in hypoxic tNMR treated cells and lowest in normoxic control cells (Figure 3I). In summary, tNMR treatment under hypoxic conditions significantly affected all measured metabolites in comparison to sham hypoxic cells, except for the intracellular ratio of NAD^+^/NADH.

### The pentose phosphate pathway (PPP) is throttled after tNMR treatment, while cell proliferation is enhanced

**Figure 4:**
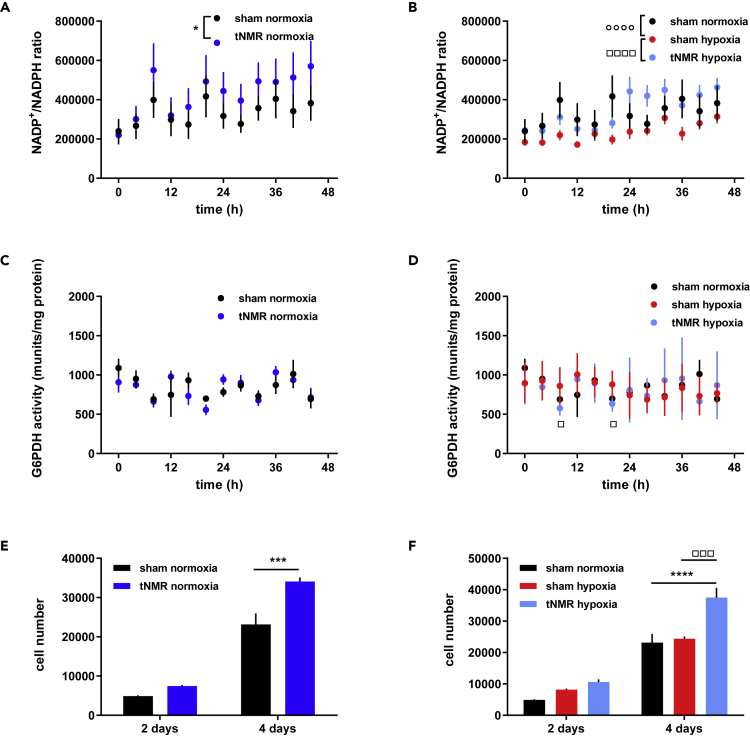
Increased NADP^+^/NADPH ratios and reduced G6PDH activities indicate a reduced flux through the PPP after tNMR treatment, while cell proliferation is increased (A) NADP^+^/NADPH ratio under normoxic conditions. (B) NADP^+^/NADPH ratio under hypoxic conditions. (C) G6PDH activity under normoxic conditions. (D) G6PDH activity under hypoxic conditions. (E) Cell numbers after two and four days under normoxic conditions. (F) Cell numbers after two and four days after hypoxic treatment; Black circles/bars represent sham treated cells, dark blue circles/bars represent ratios after treatment with tNMR, red circles/bars show levels of hypoxia sham treated cells and light blue circles/bars those of hypoxia and tNMR treated cells. Shown are means ± SEM, n = 4; asterisks mark significant differences between normoxic sham and tNMR treated cells, open circles mark significant differences between sham treated normoxic and hypoxic cells, open squares mark differences between hypoxic sham treated cells and hypoxic tNMR treated cells, calculated by two-way ANOVAs (GraphPad Prism 6.0).

To characterize the impact of tNMR on the PPP, we measured the ratios of NADP^+^/NADPH and the activity of the PPP rate limiting enzyme Glucose-6-Phosphate-Dehydrogenase (G6PDH) after normoxic and hypoxic treatment (Figure 4). Increased NADP^+^/NADPH ratios observed after the tNMR treatments indicate a relative decrease in the reduction equivalent NADPH under both oxygen tensions (Figures 4A and 4B). Sham treated cells exhibited significantly reduced ratios after the hypoxic treatment. G6PDH activity was not altered in normoxic sham treated cells, but significantly reduced after hypoxic tNMR treatment at two specific time points (Figures 4C and 4D). In summary, tNMR led to a rather reduced flux through the PPP immediately after the treatments, compared to sham treated cells. Interestingly, the decreased flux through the PPP after tNMR treatment occurred against the background of an increased cell proliferation, observable under both oxygen tensions (Figures 4E and 4F).

### tNMR under hypoxia increases mitochondrial and extracellular, but reduces cytosolic ROS

**Figure 5:**
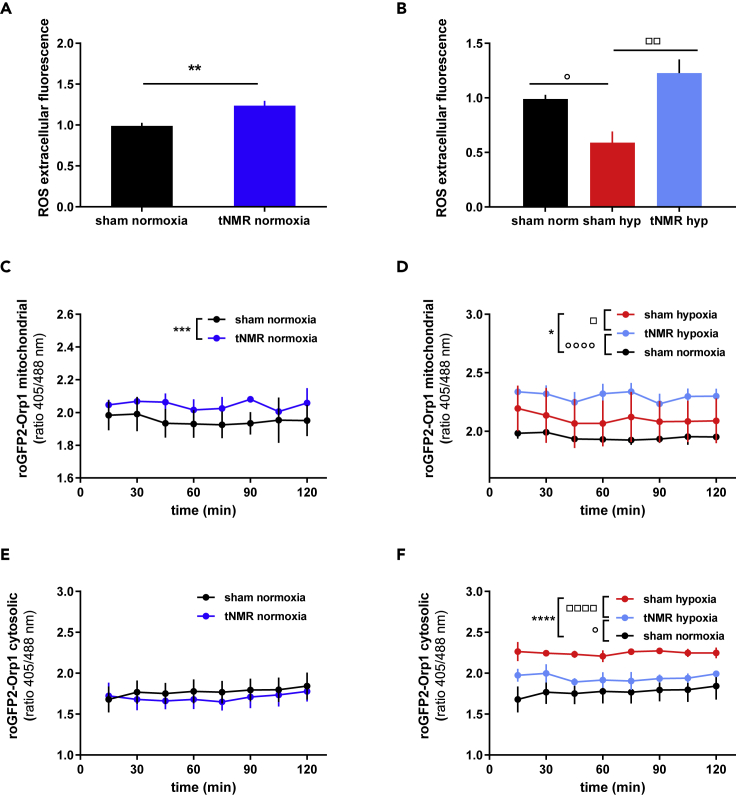
tNMR increases extracellular and mitochondrial ROS, while cytosolic ROS is reduced (A) Extracellular ROS after normoxic sham and tNMR treatment. (B) Extracellular ROS after hypoxic sham and tNMR treatment. (C) mitochondrial ROS after normoxic. (D) mitochondrial ROS after hypoxic. (E and F) cytosolic ROS after normoxic and (F) cytosolic measurement after hypoxic sham and tNMR treatment. Shown are means ± SEM; n = 3 to 5; asterisks mark significant differences between normoxic sham and tNMR treated cells, open circles mark significant differences between sham treated normoxic cells and cell treated with tNMR under hypoxic conditions, open squares mark differences between sham treated and tNMR treated cells under hypoxia, calculated by two-way ANOVAs (GraphPad Prism 6.0).

Because EMFs are known to affect ROS levels via the radical pair mechanism (RPM)^3^^,^^24^^,^^25^^,^^26^^,^^27^ we determined the levels of extracellular, mitochondrial and cytosolic ROS, more specifically the amounts of H_2_O_2_ generated (see Figure S2 for the experimental setup). tNMR led to a significant increase in extracellular ROS levels after normoxic and hypoxic treatment (Figures 5A and 5B) compared to sham cells, which produced less extracellular ROS after hypoxic incubation for six hours. Mitochondrial ROS was significantly increased after tNMR under normoxia (Figure 5C), and the treatment under hypoxic conditions further enhanced the rise in mitochondrial ROS (Figure 5D). No alterations were found in cytosolic ROS levels after normoxic tNMR treatment (Figure 5E), whereas tNMR applied under hypoxia led to cytosolic ROS levels closer to those observed for normoxic sham treated cells than to those of hypoxic cells (Figure 5F).

### tNMR under normoxic conditions reduces the extracellular acidification rate (ECAR)

**Figure 6:**
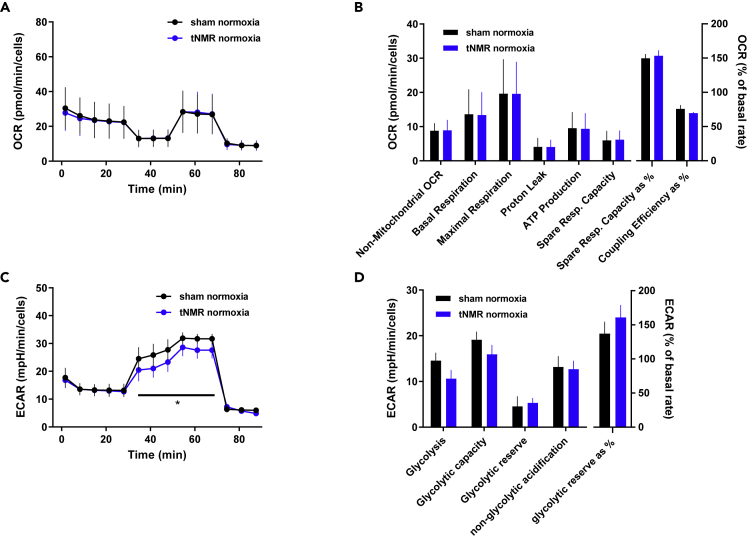
tNMR under normoxia reduces extracellular acidification rate in synchronized NIH3T3 cells (A) OCR of sham and tNMR treated cells, measurement starting immediately after treatment under normoxic conditions, (B) Non-mitochondrial O_2_ consumption, basal respiration, maximal respiration, proton leak, ATP production, spare respiratory capacity, spare respiratory capacity as a % of maximal respiration and coupling efficiency of normoxic cells. (C and D) ECAR of sham and tNMR treated cells and (D): glycolysis, glycolytic capacity, glycolytic reserve and non-glycolytic acidification of normoxic cells, shown are means ± SEM, n = 3 to 5; asterisks mark significant differences between sham and tNMR treated cells under normoxia, calculated by two-way ANOVAs (GraphPad Prism 6.0).

To further assess the metabolic phenotype of tNMR treated NIH3T3 cells we used a Seahorse Extracellular Flux Analyzer and respective mitochondrial and glycolysis stress test kits (Agilent) using the experimental setup shown in Figure S2. Normoxic tNMR treatment did not alter mitochondrial respiration (OCR) (Figures 6A and 6B), but reduced the ECAR in tNMR treated cells (Figure 6C). Though a trend towards a reduced glycolysis was visible, the changes in the glycolytic parameters measured were not significant (Figure 6D).

### Hypoxic tNMR treatment reduces ECAR and throttles the hypoxia induced increase in glycolysis

**Figure 7:**
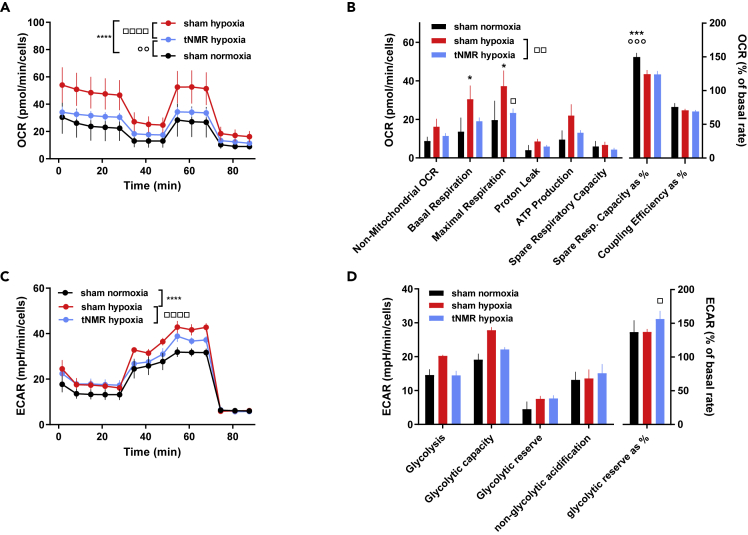
tNMR under hypoxia reduces reoxygenation induced increase in OCR and ECAR in synchronized NIH3T3 cells (A) OCR of sham and tNMR treated cells, measurement starting immediately after treatment under hypoxic conditions. (B) Non-mitochondrial O_2_ consumption, basal respiration, maximal respiration, proton leak, ATP production, spare respiratory capacity, spare respiratory capacity as a % of the maximal respiration and coupling efficiency. (C and D) ECAR of sham and tNMR treated cells and (D): glycolysis, glycolytic capacity, glycolytic reserve and non-glycolytic acidification after hypoxic treatment, expressed as a % of ECAR ; shown are means ± SEM, n = 3 to 6; asterisks mark significant differences between sham and tNMR treated cells under normoxia, open circles differences between normoxic sham cells and tNMR treated cells under hypoxia, open squares indicate differences between hypoxic sham and tNMR treated cells under hypoxia, calculated by two-way ANOVAs (GraphPad Prism 6.0).

Hypoxic sham treated cells responded to the reoxygenation with a significantly increased mitochondrial respiration (OCR), an increased basal and maximal respiration and a concomitant reduction in the spare respiratory capacity (in % of maximum respiration) (Figures 7A and 7B). Mitochondrial respiration of hypoxic tNMR treated cells resided closer to those of normoxic sham treated cells, and also basal and maximal respiration were reduced compared to hypoxic sham cells. ECAR was significantly elevated in hypoxic sham cells, concomitant with a trend towards increased glycolysis and glycolytic capacity (Figures 7C and 7D), whereas tNMR treated cells under hypoxia exhibited ECAR`s in between those of normoxic and hypoxic sham cells. Compared to hypoxic sham cells, the glycolytic reserve in % was higher in tNMR treated cells, which indicates a further capacity of tNMR treated cells to increase the glycolytic flux when needed.

## Discussion

The effects of weak EMF's on somatic cellular clocks, apart from the retinal CRY mediated magnetoreception in migrating animals, has been reported in several studies.^1^^,^^2^^,^^3^^,^^4^ Knowledge on the underlying mechanisms is still scarce, although several groups increasingly focus on underlying quantum mechanical effects such as the RPM.^2^^,^^24^^,^^25^^,^^28^ Even less known are any potential physiological consequences arising from the impact of EMF's on circadian clocks. Previously, we were able to show that tNMR did not only affect clock protein members in mammalian NIH3T3 cells, but also HIF-1α,^4^which is known to be tightly linked to the circadian clock.^5^^,^^6^ Because HIF-1α is known to regulate the adaption of cellular metabolism in response to altered oxygen tensions, we wanted to assess basic cellular metabolism after normoxic and hypoxic incubation with and without tNMR radiation, simulating therewith also a classical reoxygenation event. In a first experiment, we addressed HIF-1α mRNA and protein levels in Dex synchronized NIH3T3 cells after six hours of normoxic or hypoxic tNMR irradiation (Figure 1, for experimental setup see Figure S1). Although unaltered under normoxic conditions, tNMR treatment substantially increased mHif-1α mRNA, surpassing levels of hypoxia sham treated cells during the second day of sampling. Counter-intuitively, protein levels of HIF-1α appeared to be reduced after hypoxia, and even more after the hypoxic tNMR treatment, which we partly assign to the HIF-1α protein reducing effect of the Dex treatment, according to,^23^^,^^29^ and partly to the reoxygenation itself. Temporal expression of HIF-1α was as well significantly altered between hypoxic and hypoxic tNMR treated cells. Despite the observed decreased levels of HIF-1α protein, hypoxia treated cells elicited a canonical hypoxic response, indicated by decreased levels of glucose and pyruvate, decreased intracellular ratios of ADP/ATP and NAD^+^/NADH, whereas levels of intracellular ADP, ATP, intra- and extracellular lactate appeared to be increased (Figure 3). Irradiation with tNMR under normoxia specifically reduced ADP and lactate concentrations (Figure 2), whereas the hypoxic irradiation resulted in a metabolic signature in between normoxic and hypoxic cells by reducing all measured metabolites in comparison to hypoxic sham cells, except for the NAD^+^/NADH ratio, which remained unaltered (Figure 3).

The apparent reduced glycolytic flux after hypoxic tNMR treatment led us to investigate the role of the PPP. Under both oxygen tensions, tNMR exposure appeared to increase the intracellular NADP^+^/NADPH ratios (Figure 4), indicating a decrease in NADPH, whereas sham treated hypoxic cells exhibited significantly reduced ratios compared to normoxic sham cells, hence an increase in the reducing equivalents NADPH. In addition, no activation of the PPP via the rate limiting enzyme G6PDH was found in any of the treatment groups, quite the contrary, a slight reduction of enzyme activity at two specific time points was found in hypoxia tNMR cells. Accordingly, the results suggest that tNMR treated NIH3T3 cells did not need an increased flux through the PPP, at least after the treatment, neither for fighting unfavorable redox conditions through production of the reducing equivalent NADPH, nor for the delivery of ribose-5-phosphate for anabolic processes.

Cell proliferation rates, though, appeared to be significantly increased after tNMR treatment under both investigated oxygen tensions (Figures 4E and 4F). The influence of EMF's on cellular proliferation has been addressed repeatedly and was shown to depend on alterations in cellular ROS signaling, more specifically on mitochondrial ROS levels.^30^ However, the reports are rather inconsistent.^3^^,^^24^^,^^31^^,^^32^ Reasons for this seeming inconsistency of the correlation between EMF exposure and cell proliferation are the diverse types of EMF's used,^31^ the localization of the measured ROS (extracellular, cytosolic, mitochondrial), the nonlinear dose response relationship^4^^,^^33^^,^^34^ and most probably also the cell types investigated. Consistent with our data, increased proliferation rates after the treatment with RF EMF's were also reported by.^24^^,^^31^ In accordance with these studies on RF EMF's we also found mitochondrial as well as extracellular ROS increased (Figure 5). Interestingly, cytosolic ROS levels appeared to be reduced after the hypoxic tNMR treatment in comparison to hypoxic sham treated cells (Figure 5F). The increased cytosolic ROS levels of the latter are most probably because of the increase in mitochondrial respiration (OCR) (Figures 7A and 7B), with which the cells were compensating for the reduced oxygen availability during the six-hour treatment of tNMR under hypoxia of 1%. Hence our experiment simulates also a classical ischemia reperfusion (IR) event, which is known to perturb redox balance and to cause injury under pathophysiological conditions.^17^^,^^35^^,^^36^ tNMR applied under hypoxic conditions appeared to reduce both during reoxygenation, mitochondrial respiration as well as cytosolic ROS levels.

Increased extracellular as well as mitochondrial ROS production after EMF exposure have been shown to be generated through the spin correlated RPM. This quantum based mechanism is an accepted model which explains the modulation of spin correlated radical pair states produced by semiquinone flavin (FADH) enzymes and O_2_^-^. Magnetic field alterations thereby lead to changes in the product ratio between singlet product yield (H_2_O_2_) and triplet product yield (O_2_^⋅−^), which eventually affect the outcome of cellular ROS product ratios. The RPM was characterized in detail for the clock protein CRY, which meanwhile is an accepted magnetic receptor to enable compass orientation of migrating animals.^26^^,^^37^ In addition, CRY was shown to be responsible for the intra- as well as extracellular ROS accumulation after exposure to a pulsating electromagnetic field of 1.8mT and 10 Hz, using murine mCry1/mCry2 double knockout cells^3^ Apart from CRY, the RPM was also found to occur at the protein complexes of the mitochondrial electron transport chain,^25^ and at several ROS production entities throughout the cells which depend on Flavin-dependent enzymes^38^, like the NADPH oxidase which transports electrons from NADPH across the plasma membrane to O_2_, resulting in the production of O_2_-.^2^ Changes in the product yields of radical pairs are predicted for fields at frequencies corresponding to hyperfine couplings in the range between 1 to 100 MHz.^39^ The field intensity of 0.4 mT in combination with a RF of 17 kHz used in our study to induce water proton nuclear magnetic resonance are clearly far below that range. However, we found elevated extracellular ROS levels, which from our current data set we can neither attribute to CRY nor to NADPH oxidase activity. The observed alterations in cytosolic and mitochondrial ROS signaling, as well as in mitochondrial respiration and glycolysis remarkably resemble the RPM based effects reported by.^25^ The authors used primary human umbilical vein endothelial cells (HUVECs) and compared the effects of a single static magnetic field (MF) and a combined RF EMF on cellular bioenergetics. They found that H_2_O_2_ and O_2_^⋅−^ product yields depended on the angle of the applied RF (parallel or perpendicular) and that either OCR or ECAR of the cells were affected, depending again on the angle of the applied field. Under both situations though, either OCR or ECAR appeared to increase. Beside the different cell types used, Usselman et al.^25^ did not carry along a sham control, which means cells without any artificial MF exposure. Furthermore, our approach to study the effects of tNMR combined with two different oxygen tensions (normoxia and hypoxia of 1% O_2_) adds additional complexity for comparison. tNMR under both, normoxic and hypoxic conditions appeared to reduce ECAR by throttling glycolysis (Figures 6 and 7), whereas OCR was kept constant under normoxia , and the reoxygenation induced rise in OCR of hypoxic cells was strongly diminished. Hence, the OCR of hypoxic tNMR treated cells during reoxygenation can be interpreted as “kept as constant as possible” in comparison to hypoxic sham cells.

Because there literally are no studies on the cell physiological effects of proton nuclear resonances we can only hypothesize which molecules or processes might be directly or indirectly affected by them. Only recently, the (re) orientation of water was demonstrated to control hyperfine electronic couplings in CRY. More specifically, the hydrogen bonding between the trytophans B and C in the CRY protein, which is necessary to form an electron-tunneling route, was demonstrated to be affected by the motions of the captured single water molecule and thus to depend on the local water solvation dynamics.^40^ Apart from CRY, we assume that also mitochondrial flavoproteins, such as the Electron transfer flavoprotein or the Lipoamide dehydrogenase,^41^^,^^42^ might be affected by proton resonances. This would also explain the rise in mitochondrial ROS production we observed in tNMR treated cells under hypoxic conditions (Figure 5D).

Given that hypoxia prevails in cells and tissues of organisms and that pathophysiological reoxygenation events are known to be even more problematic than hypoxia itself after infarct and stroke,^17^^,^^35^^,^^36^ the potential of tNMR to alter cellular metabolism even under or after low oxygen tensions is probably the most important finding of the present study. The obvious potential of tNMR to modulate cellular bioenergetics might also help to explain the recently reported effects of the treatment on the regeneration of primary rat dorsal root ganglion-derived Schwann cells in vitro^43^

The role HIF-1α actually plays in the present settings, is still to question. HIF-1α is known to be directly regulated by ROS^44^ and to regulate cellular and, in particular, mitochondrial metabolism itself.^45^ In addition, HIF-1α is also known to be negatively regulated by CRY1.^46^ Against this background the observed sensitivity of HIF-1α to changes in the external MF does not seem to be that exceptional. Even a quantum based direct regulation of HIF-1α through RF EMF's in general is conceivable. As already mentioned, protein levels of HIF-1α are regulated through hydroxylation of the proline residues 402 and/or 564 in an oxygen dependent manner. tNMR might directly affect these regulatory hydroxylation events of the protein through the proton resonance conditions induced. This idea has already been proposed for the DNA repair enzyme family AlkB by.^47^ In addition, the stabilization of HIF-1α protein was shown to depend on the concentration of cellular FAD.^48^

However, metabolic reprogramming as a therapeutic tool seems promising and has already repeatedly been suggested.^28^^,^^49^^,^^50^ The increased HIF-1α driven glycolytic flux, which is commonly accompanied by increased lactate concentrations, is often a problem per se in pathophysiological conditions such as inflammation, infection (Covid-19), ischemic diseases as infarct and stroke, and also in tumor development and progression. The present study implicates that tNMR might have the potential to counteract the Warburg effect known from many cancer cells which are prone to glycolysis even under aerobic conditions. In this context, even very low doses of ionizing radiation commonly used for the treatment of tumors have been shown to increase glycolysis and lactate production,^51^which does not seem to be the case for tNMR at first sight. Hence, we strongly recommend to cautiously investigate tNMR as treatment option for pathophysiological conditions, in which a rewiring of basic cellular metabolism might be of advantage, given that no side effects of the treatment have been reported so far over the last two decades.

### Limitations of the study

The dose response relationship between electromagnetic fields and biological matter is nonlinear, as mentioned above. This necessarily means that the results presented here only refer to the applied field intensity, frequency and the duration of 6 h. A longer or shorter duration of the treatment might therefore have a completely different outcome, as already demonstrated in.^4^

## STAR★Methods

### Key resources table

**Table:** 

REAGENT or RESOURCE	SOURCE	IDENTIFIER
**Antibodies**		

Mouse monoclonal anti-HIF-1 alpha	Abcam	Cat# ab179483, RRID: AB_2732807

**Chemicals, peptides, and recombinant proteins**		

Dexamethasone	Sigma-Aldrich	D4902; CAS: 50-02-2
Hoechst 33342, (2,5′-Bi-1H-benzimidazole, 2'-(4-ethoxyphenyl)-5-(4-methyl-1-piperazinyl)- 23491-52-3)	Thermo Fisher Scientific	H1399; CAS: 23491-52-3
Gas mixture (CO_2_ 5%; O_2_ 21%;74% N_2_); 50L	Air Liquide	1905.600.151
Gas mixture (CO_2_ 5%; O_2_ 1%; 94%N_2_); 50L	Air Liquide	1909.260.151

**Critical commercial assays**		

Amplex™ Red Hydrogen Peroxide/Peroxidase Assay Kit	Thermo Fisher Scientific	A22188
Glucose Assay Reagent	Sigma-Aldrich	G3293
L-LDH	Sigma-Aldrich	L-2500
ADP/ATP Ratio Assay KIT	Sigma-Aldrich	MAK135-1KT
NADP/NADPH-Glo™ Assays	Promega	G9082
Glucose-6-phosphat DH Activity Assay Kit	Sigma-Aldrich	MAK015
Seahorse XFp Cell Mito Stress Test Kit	Agilent technologies	103010–100
Seahorse XFp Glycolysis Stress Test Kit	Agilent technologies	103017–100

**Experimental models: Cell lines**		

Mouse: NIH 3T3 cells	ATCC	CRL-1658

**Oligonucleotides**		

Primer mHif-α F: 5`-GAG TCT GAA GTT TTT TAT GAG CTT GCT-3′ R:5` -GGT GAG CCT CAT AAC AGA AGC TTT-3′	This paper	N/A

**Recombinant DNA**		

Mammalian: cytosolic roGFP2-Orp1 (retroviral vector)	Gutscher et al. J Biol Chem. 2009 Nov 13;284(46):31532-40^54^	Addgene Plasmid #64991
Mammalian: mitochondrial roGFP2-Orp1 (retroviral vector)	Gutscher et al. J Biol Chem. 2009 Nov 13;284(46):31532-40^54^	Addgene Plasmid #64992

**Software and algorithms**		

GraphPadPrism 6	GraphPad Software Inc	https://www.graphpad.com/scientific-software/prism/
BioRender	BioRender	https://biorender.com/
Cosinor Analysis Algorithm: *Y* = ((*m*×*X*) + *C*)+ amplitude × exp(–*k* × *X*)×cos(((2 × pi × (*X*-phase))/period))	GraphPad Software Inc	https://www.graphpad.com/scientific-software/prism/

### Resource availability

#### Lead contact

Further information and requests for resources and reagents should be directed to and will be fulfilled by the lead contact, Margit Egg (margit.egg@uibk.ac.at).

#### Materials availability

All requests for resources or reagents should be directed to and will be fulfilled by the lead contact author.

#### Data and code

• All data reported in this paper will be shared by the lead contactupon request.
• This paper does not report original code.
• Any additional information required to reanalyze the data reported in this paper is available from the lead contact upon request.

### Experimental model and subject details

For all experiments the mouse fibroblast cell line NIH3T3 (LGC Standards, USA) was used. Culture of the cells was performed in Dulbecco’s Modified Eagle’s Medium (Life Technologies, USA), supplemented with 10% Calf Serum (Sigma Aldrich, USA) and 1% Penicillin-Streptomycin (Life Technologies, USA) at 37°C under constant darkness at 5% CO_2_ in a humidified incubator.

### Method details

#### Experimental setup and sampling

NIH3T3 cells were cultured as outlined in.^4^Due to better working conditions, cells were seeded under dim light, but kept strictly without any additional light exposure during the subsequent procedures. Synchronization of the NIH3T3 cellular clocks was achieved via Dexamethasone (Dex, Sigma Aldrich, USA) treatment, as described in.^52^ Briefly, cells were incubated in Dex (Sigma Aldrich, USA) for three hours at a final concentration of 100 nM. tNMR exposures were conducted for 6 h using the MBST®-Open System 350 (MedTec Medizintechnik GmbH, Germany), with a field intensity of 0.4 mT and a radio frequency of 17 kHz. Sham as well as tNMR treatments were performed at the same time, under the same conditions, the sham group being exposed at a distance of 2 m from the tNMR device. Both treatments were performed under normoxic (5% CO_2_, 21% O_2_, 74% N_2_) or hypoxic conditions (5% CO_2_, 1% = O_2_, 94% N_2_), using commercial gas mixtures (Airliquide Deutschland GmbH, Germany). The temperature of the whole treatment chamber was adjusted using a temperature sensor that held the experimental conditions precisely at 37°C. Timeline of the experimental treatments are depicted in Figure S1.

#### Measurement of metabolites

Metabolic measurements were performed as described in.^53^ Extracellular measurements were taken from the cells'media, collected in parallel to the metabolite samples. For the intracellular sampling, cells were washed with 1xPBS, resuspended in 850μL of Tris/EDTA buffer (100 mM Tris, 4 mM EDTA, pH 7.75), scraped off and flash frozen in liquid nitrogen. Complete cell lysis was implemented trough subsequent up and down pipetting. Lysates were centrifuged for 1 min at 5000 rcf at 4°C. For normalization of the metabolite data, 20μL aliquots of the supernatants were frozen to determine the total protein contents, using the NanoDrop™ 2000/2000c Spectrophotometer (Thermo Scientific, Germany). Residual samples were deproteinated via a 15-min centrifugation step at 14000 rcf using 10kD molecular weight cut off centrifugal filters (Merck, Germany). Steady state levels of intracellular glucose were determined with the Glucose HK Assay Reagent kit (Sigma-Aldrich, Germany). Lactate, pyruvate and the resulting calculated intracellular NAD+/NADH ratio were measured using the innate fluorescence of NADH versus NAD+, as described in detail in.^53^ NAD+/NADH ratios were calculated from lactate and pyruvate data with the formula: pyruvate/lactate∗9000 ([NAD+)/[NADH] = [pyr]/[lac]∗10ˆ-pH/Kequ; Kequ of LDH = 1.11∗10–11; pH7). ADP/ATP ratios were determined with the ADP/ATP ratio Assay Kit (Sigma-Aldrich, Germany), NADP+/NADPH ratios were determined with the NADP/NADPH-Glo™ Assay Kit (Sigma-Aldrich, Germany), activity of Glucose-6-Phosphate Dehydrogenase (G6PDH) was measured using the Glucose-6-Phosphate Dehydrogenase Assay Kit (Sigma-Aldrich, Germany). All measurements were performed in 96-well plates, using transparent tissue culture plates (Sarstedt, Austria) for glucose concentrations and G6PDH activity and white plates (Sarstedt, Austria) for the bioluminescence (ADP/ATP ratios, NADP+/NADPH ratios) and the fluorescence measurements (lactate and pyruvate). Reading was performed in a Victor X4 2030 Multilabel Reader (PerkinElmer, Germany), values were normalized to the amount of protein in the samples.

#### Quantitative realtime – PCR

Isolation of total RNA and cDNA synthesis of NIH3T3 cells was performed as described in.^4^ m*Hif1- α* forward and reverse primers were designed via Primer Express Software 3.0 (Applied Biosystems, USA): *mHif1- α*: accession numberNM_001313919.1 (forward (f): 5ʹ-GAG TCT GAA GTT TTT TAT GAG CTT GCT-3ʹ, reverse (r): 5ʹ-GGT GAG CCT CAT AAC AGA AGC TTT-3ʹ). Primer concentrations were optimized through a primer matrix testing the combinations of three different primer concentrations each, followed by dissociation curves to test for the product yield and the presence of unwanted products such as primer dimers. For absolute quantification of *mHif-1α* mRNA calibration curves were generated as outlined in.^4^ cDNA samples were measured in a QuantStudio™3 Real-Time PCR System using the Power SYBR® Green Master Mix (Thermo Fisher Scientific, UK). CT-values were calculated reciprocally to log10, according to the respective gene calibration curves, to achieve the absolute copy numbers. Absolute copy numbers were finally normalized to 10 ng of total RNA.

*mHif1- α*: accession numberNM_001313919.1 (forward (f): 5ʹ-GAG TCT GAA GTT TTT TAT GAG CTT GCT-3ʹ, reverse (r): 5ʹ-GGT GAG CCT CAT AAC AGA AGC TTT-3ʹ);

#### Western Blot

NIH3T3 cells were harvested in 850μL lysis buffer containing 25% glycerol, 430 mM NaCl, 1.5 mM MgCl_2_ hexahydrate , 0.2 mM Ethylenediamine tetra acetic acid disodium salt dihydrate and 40 mM HEPES, with an adjusted pH of 7.9. Protease inhibitors (1 mM Sodium Vanadate, 0.5 mM PMSF, 1 μg/mL Aprotinin, 1 μg/mL Leupeptin, 1 μg/mL Pepstatin, 0.5 mM DTT and 10 mM MG132 were added. Lysates were centrifuged at 2500rcf for 5minat 4°C. Pellets were resuspended in 50μL 2× Laemmli Sample Buffer (BioRad Laboratories, Hercules, California, USA) and heated to 95°C for 5 min. Protein concentrations were determined using the Nanodrop™ 2000c Spectrophotometer (Thermo Fisher Scientific, Waltham, Massachusetts, USA) with 20 μg of total protein used for each sample.

Proteins were separated on a 12% SDS-PAGE using BioRad Criterion™ TGX Stain-Free™ Precast Gels (BioRad Laboratories, Hercules, California, USA) and transferred to an Immun Blot® PVDF membrane for Protein Blotting (BioRad Laboratories, Hercules, California, USA) with a Trans-Blot® Turbo™ Transfer System (Bio-Rad Laboratories, Hercules, California USA). Unspecific protein-binding sites were blocked in 5% low fat powdered milk (Carl Roth GmbH, Germany). Subsequently, protein samples were dissolved in blocking buffer (50 mM TRISBase, 150 mM NaCl, pH 7.5, containing 0.1% Tween 20) for 45minat room temperature. As primary antibody, Hif1 1:1000 (ab179483, Abcam, UK) was used for overnight incubation at 4°C in blocking buffer. After three additional washing steps, the membrane was incubated for 60 min in blocking buffer at room temperature with goat anti-rabbit IgG peroxidase 1:10000 (ab6721, Abcam, USA) as second antibody. Three washing steps were carried out prior to chemiluminescent detection using the ChemiDoc™ XRS + W System (BioRad Laboratories, Hercules, California, USA) and the Amersham™ ECL™ Detection Reagent (Cytiva, Marlborough, Massachusetts, USA). Relative quantification of protein levels was accomplished by Image Lab™ Software 6.1.1 (BioRad, Germany). Determination of total protein amounts blotted on the membranes was performed using the stain free staining protocol of the ChemiDocTM XRS + W System, selecting the application “StainFree Blot”, after activation by UV irradiation of the membrane for 2.5 min. Individual HIF-1α Western blots, UV detected total protein gels, as well as overall total protein loading controls presented as means ± SEM (n = 4) are shown in Figures S3A–S3C.

#### Extracellular ROS measurement

For the fluorometric detection of extracellular H_2_O_2_ we used the Amplex ™ Red Hydrogen Peroxide /Peroxidase Assay Kit (A22188, Thermo Fisher Scientific, UK) and a Victor X4 2030 Multilabel Reader (PerkinElmer, Germany). NIH3T3 cells were seeded in 96-well plates, at a total concentration of 15000 cells per well, in 200 μL of Dulbecco’s Modified Eagle’s Medium DMEM (Life Technologies, USA) supplemented with 10 % Calf Serum (Sigma-Aldrich, USA) and 1% Penicillin-Streptomycin (Life Technologies, USA). Cells were synchronized with Dex the following day and held in a humidified cell incubator at 37°C, 5% CO_2_ until tNMR treatment. After treatment, the medium was removed and cells were washed gently with 1xPBS. Subsequently, cells were incubated in Agilent Seahorse XF Base Medium, without Phenol Red (Agilent Technologies, USA), 1.3% CS, 0.2 units/mL horseradish peroxidase, and 10 μM Amplex™ Red for two hours and then immediately detected, according to.^3^

#### Intracellular ROS measurements

NIH3T3 cells with stable expression of mitochondrially targeted or non-targeted (= cytosolic) variants of the H_2_O_2_ sensor roGFP2-Orp1 were produced by lentiviral transfection using a pLVXEP backbone. Original plasmids were a kind gift from Dr. Tobias Dick, Heidelberg, Germany, and are now available at Addgene, plasmids #64991 and #64992 . Lentiviral transfection was performed with support from Dr. Jerome Mertens, Institute of Molecular Biology, University of Innsbruck. The roGFP2-Orp1 expressing cells were seeded onto black 96 well plates with clear bottom (Perkin Elmer, Germany) at a density of 15000 cells per well in 200 μL of Dulbecco’s Modified Eagle’s Medium DMEM (Life Technologies, USA) supplemented with 10% Calf Serum (Sigma-Aldrich, USA) and 1% Penicillin-Streptomycin (Life Technologies, USA). Cells were synchronized with Dex the following day and held in a humidified cell incubator at 37°C, 5% CO_2_ until tNMR treatment. After the treatment, cells were immediately washed with prewarmed Hanks buffered salt solution adjusted to pH 7.4 to remove the remaining growth media and were incubated with 200 μL of the buffer per well for subsequent ratiometric fluorescence measurement (EX1/EM1 490 nm/515 nm and EX2/EM2 405 nm/515 nm) in an Enspire multilabel platereader (PerkinElmer, Germany). Cells treated with the oxidant Menadione (100 μM f.c.) or the antioxidant Dithiothreitol (1 mM f.c.) served as fluorescent read-out positive or negative control, respectively. The measurement was conducted every 15 min for two hours.

#### Cellular respiration and acidification

Oxygen consumption rate (OCR) and extracellular acidification rate (ECAR) in NIH3T3 cells were measured with a Seahorse XFp Analyzer (Agilent, USA). Cells were seeded at a density of 5000 cells in 100 μL of Dulbecco’s Modified Eagle’s Medium DMEM (Life Technologies, USA) supplemented with 10% Calf Serum (Sigma-Aldrich, USA) and 1% Penicillin-Streptomycin (Life Technologies, USA). Cells were synchronized with Dex the following day and held in a humidified cell incubator at 37°C, 5% CO_2_ until tNMR treatment. Immediately after the treatment cells were washed with XF Base medium (Agilent, USA) supplemented with 2 mM L-glutamine (2 mM) or glucose (5.5 mM), pyruvate (1 mM) and L-glutamine (2 mM), for Glycolysis stress test or Mitochondrial stress test, respectively. The tests were performed according to the instructions provided by the manufacturer using the commercially available Seahorse XFp kits (Agilent, USA). Glucose and inhibitors for the Glycolysis stress test were applied in the following final concentrations: glucose (10 mM), Oligomycin (1.5 μM) and 2-deoxyglucose (2DG, 50 mM). Inhibitors for the Mitochondrial stress test were applied in the following final concentrations: Oligomycin (1.5 μM), Carbonyl cyanide-p-trifluoromethoxyphenylhydrazone (FCCP, 1 μM), Rotenone and Antimycin A (RotAMA, 0.5 μM each). Rate data from the XF assays was normalized to cell density obtained after each Seahorse run by subsequent staining of cells in the cell cartridges with Hoechst 33342 nuclear stain (f.c. 10 μg/mL; Sigma Aldrich) and fluorescent measurement in a plate reader (Victor X4 2030 Multilabel Reader). Rate data was also transformed into metabolic parameters as stated in the respective user manuals provided by the manufacturer using Microsoft Excel.

### Quantification and statistical analysis

Data are presented as mean ± SEM. The comparison between two means was conducted via Student’s t-test (two-tailed). The comparison of more than two means was performed via the two-way analysis of variance (ANOVA), followed by Holm-Sidak *post-hoc* test. In case of non-parametric variance analysis, so that the normality failed, the Kruskal-Wallis one-way ANOVA was utilized. The significance level was set at p ≤ 0.05. Cosinor analysis were performed as outlined in (Oliva et al., 2019) using Graph Pad Prism 6 (GraphPad Software, USA).
