# Supplementary material for: Quantum based effects of therapeutic nuclear magnetic resonance persistently reduce glycolysis
Source: iScience. 2022 Nov 9;25(12):105536. doi: 10.1016/j.isci.2022.105536 (PMC9700021; doi:10.1016/j.isci.2022.105536)

iScience, Volume *25*

Supplemental information

Quantum based effects of therapeutic nuclear magnetic resonance persistently reduce glycolysis

Viktoria Thöni, David Mauracher, Anil Ramalingam, Birgit Fiechtner, Adolf Michael Sandbichler, and Margit Egg

Supplemental Figure S1: Experimental setup, related to **Figures 1, 2, 3, 4 (A to D)** of the main text

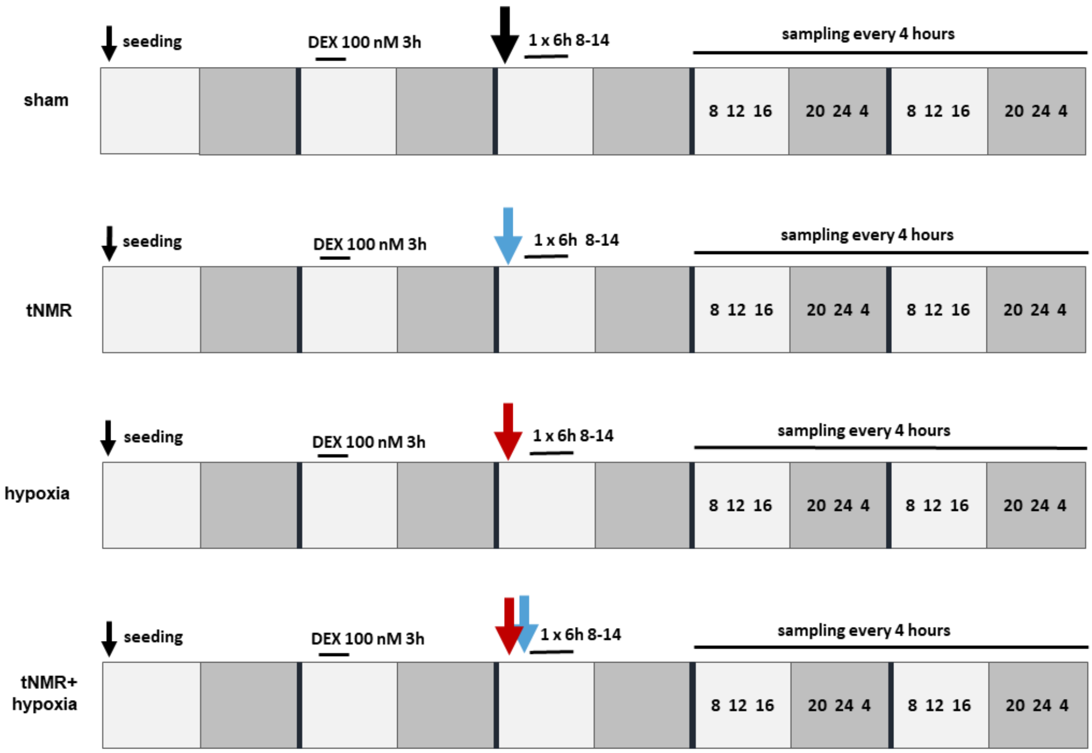

Supplementary Figure S2: Experimental setup used for ROS and seahorse measurements shown in **Figures 5, 6 and 7** of the main text.

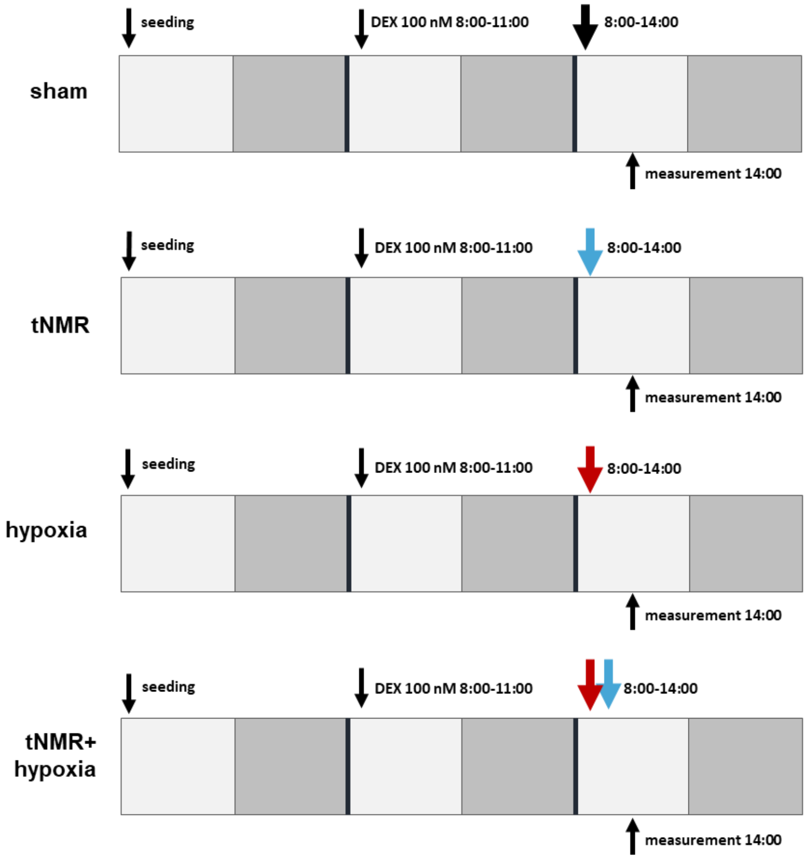

Supplemental Figure S3: (A) HIF-1α western blots, **(B)** UV detected total protein and **(C)** quantification of total protein, related to STAR methods (section Western blots)

A

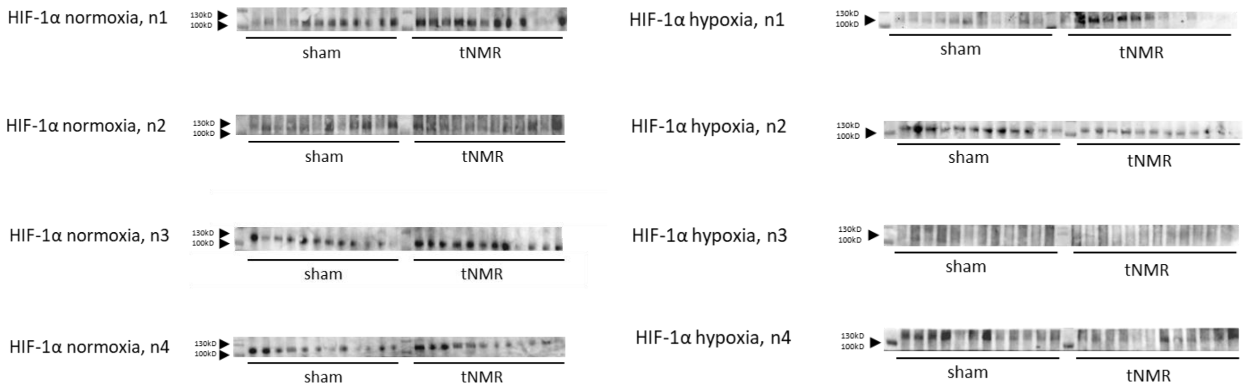

To normalize the HIF-1α raw protein data to be between 0 and 1, the following formula was used:

$$z_i = (x_i - \min(x)) / (\max(x) - \min(x))$$

where:

zi: The ith normalized value in the dataset

xi: The ith value in the dataset

min(x): The minimum value in the dataset

max(x): The maximum value in the dataset

B

total UV detected protein, normoxic samples | total UV detected protein, hypoxic samples

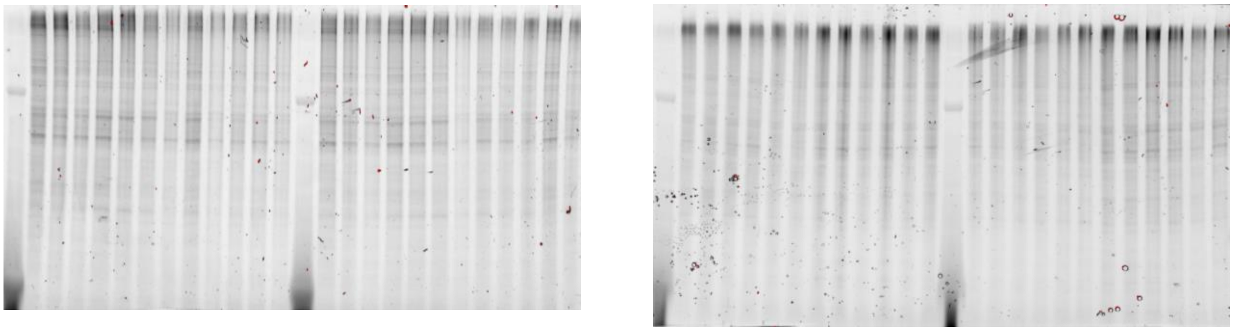

C

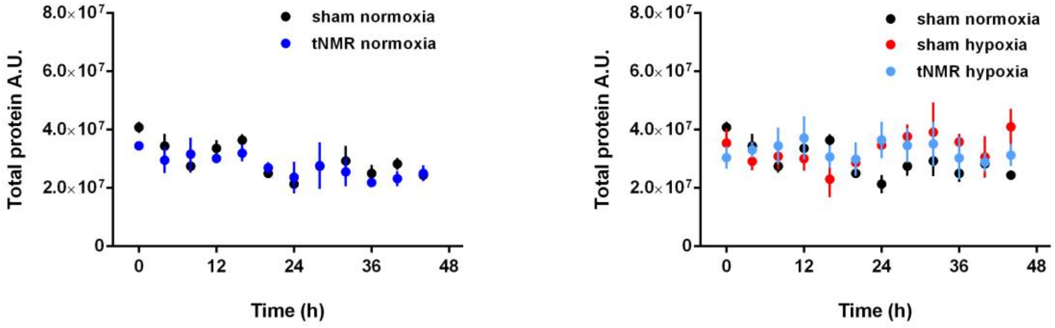

Supplement: Document S1. Figures S1–S3 [file mmc1.pdf]
